# Steviol Reduces MDCK Cyst Formation and Growth by Inhibiting CFTR Channel Activity and Promoting Proteasome-Mediated CFTR Degradation

**DOI:** 10.1371/journal.pone.0058871

**Published:** 2013-03-11

**Authors:** Chaowalit Yuajit, Sureeporn Homvisasevongsa, Lisa Chatsudthipong, Sunhapas Soodvilai, Chatchai Muanprasat, Varanuj Chatsudthipong

**Affiliations:** 1 Department of Physiology, Faculty of Science, Mahidol University, Bangkok, Thailand; 2 Division of Physical Science, Faculty of Science and Technology, Huachiew Chalermprakiet University, Samutprakarn, Thailand; 3 Research Center of Transport Protein for Medical Innovation, Faculty of Science, Mahidol University, Bangkok, Thailand; University of Geneva, Switzerland

## Abstract

Cyst enlargement in polycystic kidney disease (PKD) involves cAMP-activated proliferation of cyst-lining epithelial cells and transepithelial fluid secretion into the cyst lumen via cystic fibrosis transmembrane conductance regulator (CFTR) chloride channel. This study aimed to investigate an inhibitory effect and detailed mechanisms of steviol and its derivatives on cyst growth using a cyst model in Madin-Darby canine kidney (MDCK) cells. Among 4 steviol-related compounds tested, steviol was found to be the most potent at inhibiting MDCK cyst growth. Steviol inhibition of cyst growth was dose-dependent; steviol (100 microM) reversibly inhibited cyst formation and cyst growth by 72.53.6% and 38.2±8.5%, respectively. Steviol at doses up to 200 microM had no effect on MDCK cell viability, proliferation and apoptosis. However, steviol acutely inhibited forskolin-stimulated apical chloride current in MDCK epithelia, measured with the Ussing chamber technique, in a dose-dependent manner. Prolonged treatment (24 h) with steviol (100 microM) also strongly inhibited forskolin-stimulated apical chloride current, in part by reducing CFTR protein expression in MDCK cells. Interestingly, proteasome inhibitor, MG-132, abolished the effect of steviol on CFTR protein expression. Immunofluorescence studies demonstrated that prolonged treatment (24 h) with steviol (100 microM) markedly reduced CFTR expression at the plasma membrane. Taken together, the data suggest that steviol retards MDCK cyst progression in two ways: first by directly inhibiting CFTR chloride channel activity and second by reducing CFTR expression, in part, by promoting proteasomal degradation of CFTR. Steviol and related compounds therefore represent drug candidates for treatment of polycystic kidney disease.

## Introduction

Polycystic kidney disease (PKD) is an inherited disorder characterized by the presence of enlarging fluid-filled cysts, which disrupt the normal renal parenchyma and eventually leads to end-stage renal failure [1], [2]. Autosomal dominant polycystic kidney disease (ADPKD), the most common form of PKD, is caused by mutation of PKD1 or PKD2, which encode the proteins polycystin-1 or polycystin-2, respectively [3]. The majority of ADPKD patients require kidney replacement or renal hemodialysis [4]. Currently there is no specific pharmacotherapy for this disease.

Although the exact mechanism of ADPKD pathogenesis is not known, studies have shown that an increase in cAMP level within the renal epithelial cells lining the cyst plays a central role in PKD cystogenesis. The increase in intracellular cAMP level stimulates renal epithelial cell proliferation and raises transepithelial fluid secretion into the cyst lumen [2], [5], [6]. This fluid secretion is driven by cAMP-activated transepithelial chloride transport via the cystic fibrosis transmembrane conductance regulator (CFTR) chloride channel located at apical membrane (facing the lumen) of the cells lining the cyst [7]. Intracystic accumulation of chloride draws sodium and water into the cyst cavity via a paracellular pathway [8]. Inhibition of CFTR chloride channel with CFTR inhibitors has been shown to slow down cystogenesis both in an *in vitro* Madin-Darby canine kidney (MDCK) cell culture model and an *in vivo* model of PKD [9], [10]. Therefore, CFTR chloride channel has been proposed as a potential target for PKD intervention.

Stevioside, extracted from *Stevia rebuadiana*, is widely used as a noncaloric sweetener in food in several countries in Asia and South America [11]. The pharmacokinetics of stevioside show that it is first converted to the major metabolite steviol by intestinal microflora before being absorbed in the intestine and distributed by the blood to several organs, including intestine, liver, and kidney [12], [13], [14]. Previous studies reported that steviol underwent both phase I and phase II metabolism, with steviol glucuronide being detected as a major metabolite in the blood [11]. Most consumed stevioside is excreted as steviol glucoronide in urine [11]. The reported therapeutic properties of stevioside include anti-inflammatory [15], anti-hypertensive [16], anti-hyperglycemic [17], and anti-diarrheal effects [18]. In addition, steviol was found to interact with renal organic anion transporters, making it helpful in enhancing the therapeutic efficacy of drugs [19], [20]. It is of interest to note that steviol and its derivative (dihydroisosteviol) inhibit cAMP-activated chloride secretion by targeting CFTR in a human colonic epithelial cell line [18]. Thus, it appeared possible that steviol and its derivatives could inhibit the CFTR chloride channel in PKD.

In the present study, we investigated the inhibitory effects and mechanism of action of steviol and its derivatives on cyst formation and enlargement in the MDCK cell model of PKD.

## Materials and Methods

### Cell culture

Type I MDCK epithelial cell line, kindly provided by Professor David N. Sheppard (University of Bristol, Bristol, UK), was cultured at 37 °C under a humidified atmosphere of 5% CO_2_/95% O_2_ in a 1∶1 mixture of Dulbecco's modified Eagle medium (DMEM) and Ham's F-12 nutrient medium supplemented with 10% fetal bovine serum (FBS), 100 U/ml penicillin, 100 µg/ml streptomycin, and 5 µg/ml insulin-transferin-selenium-X supplement [21]. The solvent used for preparing steviol was dimethyl sulfoxide (DMSO). Concentration of DMSO in all experiments was less than 0.5% v/v.

### Chemicals and reagents

Steviol, isosteviol, dihydroisosteviol, and 16-oxime isosteviol were synthesized as described previously [18]. The purity of all compounds was checked by thin layer chromatography and nuclear magnetic resonance spectroscopy. Trypsin, FBS, penicillin, and streptomycin were purchased from HyClone (Waltham, MA, U.S.A.); amphotericin B, amiloride, forskolin, 8-Br cAMP, CFTR_inh_-172, protease inhibitor cocktail, and DMEM/Ham F-12 from Sigma-Aldrich (St. Louis, MO, U.S.A.); GlyH-101 [22], MG-132, propidium iodide, and (BrdU) cell proliferation assay kit from Calbiochem (San Diego, CA, U.S.A.); collagen from PureCol, Inamed Biomaterials (Fremont, CA, USA); and Annexin V-fluorescein-conjugate from Beckman coulter (Marseille, France). Rabbit and mouse anti-CFTR antibodies for western blot and immunofluorescence experiments were from Cell Signaling (Beverly, MA, USA) and Abcam (Cambridge, MA, USA), respectively. The former CFTR antibodies recognize the amino acids near the N-terminus of first transmembrane domain of CFTR, while the latter recognize amino acid residues 113–117 in the first extracellular transmembrane domain of CFTR.

### Cell viability assay

MTT (3-(4,5-dimethylthiazole-2-yl)-2,5-diphenyltetrazolium bromide) assays were used to assess the effects of steviol and its derivatives on cell viability [23]. In brief, MDCK cells (1×10^4^cells/well), seeded in 96-well plate, were grown for 24 h, and then incubated with test compounds at various concentrations for 24 and 72 h, respectively. Adherent cells were treated with serum-free MDCK media containing 10% MTT solution for 4 h under humidified atmosphere at 37 °C. Following removal of media 100 µl aliquot of DMSO was added and absorbance at 530 nm was measured. Cell viability was calculated as percent of the control group.

### MDCK cyst model

MDCK cells (800 cells/well) were suspended in 0.4 ml of 3.0 mg/ml ice-cold collagen supplemented with 10% 10X minimum essential medium (MEM), 10 mM HEPES, 27 mM NaHCO_3_, 100 U/ml penicillin, and 100 µg/ml streptomycin (pH 7.4) in a 24-well culture plate and incubated at 37 °C for 90 min in water bath to allow gelation of collagen. Then, a 1.5 ml aliquot of MDCK medium containing 10 µM forskolin was added to each well and the plate was maintained at 37 °C under humidified atmosphere of 5% CO_2_/95% O_2_. It has been well established that in the presence of forskolin in the culture medium, MDCK cells seeded onto collagen gels undergo a cAMP-dependent cell proliferation and fluid secretion as observed in ADPKD-derived cysts [24], [25].

For cyst formation experiments, test compounds were added to MDCK cell cultures in the continued presence of 10 µM forskolin from day 0. MDCK media containing forskolin and test compounds were changed every two days. At day 6, the numbers of cysts (with diameters >50 µm) and non-cyst cell colonies were counted using an inverted phase contrast light microscope (Nikon TE 2000-S, Nikon Corporation, Tokyo, Japan) at ×10 magnification. Four wells per culture condition were measured. For the index of cyst formation, percent of cyst colonies was calculated by multiplying the ratio of numbers of cyst colonies (diameter ≥50 µm) and the number of cyst plus non-cyst colonies (diameter <50 µm) by 100. Total number of colonies (cyst and non-cyst) in various conditions was around 200–300.

For determination of cyst growth, the cysts at day 6 were incubated for another 6 consecutive days. Photographs of individual cysts (the same cyst in collagen gel identified by marking on the plate) were taken at every two days (day 6, 8, 10, and 12) after seeding. To determine cyst size, the outer diameters of cysts were measured using Image J software. For inclusion in this study, each culture had to have at least 30 cysts with diameters >50 µm.

### Cell proliferation assay

MDCK cells (8×10^3^cells/well) were seeded and grown for 24 h in a 96-well plate in DMEM/Ham's F-12 media supplemented with 10% FBS and insulin-transferrin-selenium X solution (ITS) under humidified atmosphere of 5% CO_2_/95% O_2_. Adherent cells were incubated with incubation media containing 100 µM 8-Br cAMP in 0.002% FBS without ITS in the presence or absence of 100 µM steviol for 24 h. BrdU reagent solution was added at 18 h after addition of 8-Br cAMP. Absorbance at 450 nm was measured and cell proliferation is reported as percent of the OD_450_ value of the control group [23].

### Cell apoptosis assay

MDCK cells (2×10^5^) were treated with vehicle (0.1% DMSO) or 100 µM steviol for 24 h as described above. MDCK cells were suspended in 500 µl of Annexin V binding buffer (100 mM HEPES-NaOH, pH 7.4, 1.5 M NaCl, 50 mM KCl, 10 mM MgCl_2_, 18 mM CaCl_2_ in distilled water) and incubated with 0.25 µg/ml Annexin V-fluorescein conjugate and 5 µg/ml propidium iodide for 15 min at 4 C before flow cytometry analysis (BD Biosciences, San Jose, CA, USA) [26]. Cell apoptosis was quantified as the percent of the total cells undergoing apoptosis.

### Ussing chamber experiment

MDCK cells (5×10^5^cells/well) were seeded on Snapwell inserts. MDCK media were changed every two days and transepithelial resistance (R_t_) was measured using an epithelial voltohmmeter (World Precision Instruments, Sarasota, FL) as previously described [21]. On day 8, media from the apical side of MDCK cell monolayer were removed to form an air-liquid interface to enhance CFTR expression in MDCK epithelia [27]. On day 10, only MDCK polarized epithelia monolayers with resistance >2,000 Ohm.cm^2^ were used for subsequent Ussing experiments.

For short-circuit current measurements, a Snapwell insert containing MDCK cells was mounted in the Ussing chamber. Both hemichambers were filled with a Krebs' buffer solution containing 120 mM NaCl, 25 mM NaHCO_3_, 3.3 mM KH_2_PO_4_, 0.8 mM K_2_HPO_4_, 0.5 mM MgCl_2_, 10 mM HEPES, and 10 mM glucose (pH 7.4). The solution was continuously bubbled with 5% CO_2_/95% O_2_ gas mixture at 37 °C.

For apical chloride current measurements, the basolateral membrane was permeabilized with amphotericin B (250 µg/ml) for 20 min. The hemichamber at the basolateral side was filled with a high chloride buffer solution containing 140 mM NaCl, 5 mM KCl, 0.36 mM K_2_HPO_4_, 0.44 mM KH_2_PO_4_, 1.3 mM CaCl_2_, 0.5 mM MgCl_2_, 10 mM HEPES, and 4.2 mM NaHCO_3_, pH 7.2. The buffer solution facing the apical membrane had a composition similar to that of the basolateral bathing solution, except that 133.3 mM Na-gluconate, 2.5 mM NaCl, and 5 mM K-gluconate were included to generate a chloride concentration gradient from the basolateral to the apical side. Both hemichambers were connected via KCl Agar Bridge to voltage and current Ag/AgCl electrodes and clamped at 0 mV. Short-circuit current was recorded continuously using a DVC-1000 voltage clamp (World Precision Instruments, Sarasota, FL). Data were digitized using PowerLab data acquisition system (ADInstruments Inc. Colorado Springs, CO). Peak forskolin-stimulated current was recorded for analysis of steviol effect on CFTR-mediated chloride secretion.

### Western blot analysis

Cells grown in 6-well plates were incubated with lysis buffer (50 mM Tris-HCl pH 7.4, 150 mM NaCl, 1 mM EDTA, 1% Triton X-100, 1 mM NaF, 1 mM Na_3_VO_4_, 1 mM PMSF (phenylmethylsulfonyl fluoride), and protease inhibitor (PI) cocktail for 20 min at 4 °C. After centrifugation at 10,000 g for 20 min, supernatant proteins (30 µg) were separated by 12% SDS-polyacrylamide gel electrophoresis, transferred onto a nitrocellulose membrane and CFTR in MDCK cells was immunochemically detected using primary rabbit polyclonal anti-CFTR and secondary horseradish peroxidase-conjugated goat anti-rabbit IgG antibodies. The intensity of immunoreactive band is reported relative to that of β-actin (used to normalize gel loading).

### Immunofluorescence experiment

MDCK cells were grown as non-polarized epithelia on glass slides and incubated with or without 100 µM steviol for 6 h and 24 h periods. Cells were fixed in 10% methanol and incubated with primary anti-CFTR mouse monoclonal (at 1∶100 dilutions) and Alexa Fluor 568-conjugated goat anti-mouse secondary antibody (at 1∶100 dilutions). Glass slides were fixed and nucleus were stained with TOPRO-3 (at 1∶300 dilutions) and examined by a confocal laser microscopy (FV-1000; Olympus) at ×40 magnification. The data were analyzed as percent fluorescence intensity of 35 random regions of interest (ROI).

### Statistical analysis

Results of all experiments are shown as the mean±S.E.M. Statistical significance between control and treatment groups was calculated using student's unpaired *t* test, one-way ANOVA followed by Bonferroni's post hoc test or repeated measure ANOVA, where appropriate. A *P*-value of <0.05 is considered as statistically significant.

## Results

### Inhibitory effect of steviol and its derivatives on MDCK cyst formation and growth

**Figure 1:**
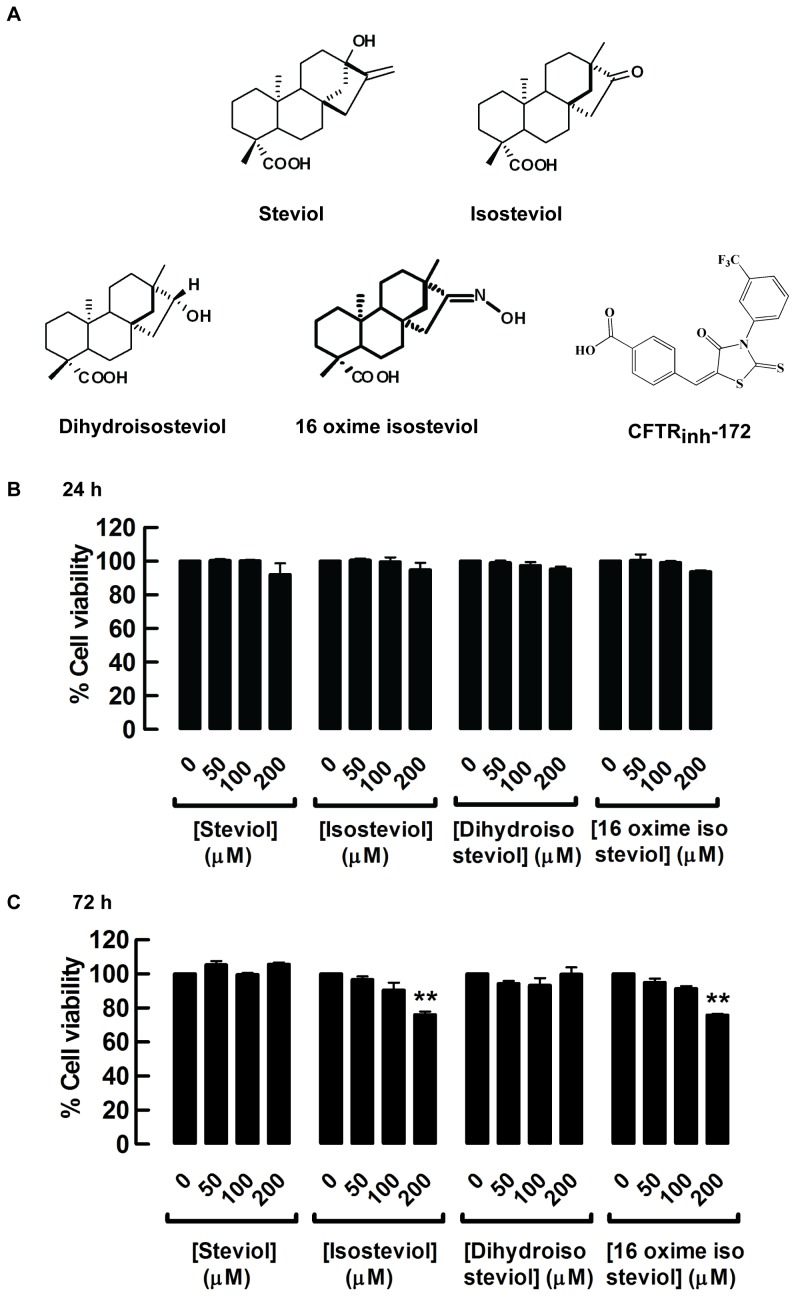
Cytotoxicity evaluation of steviol and derivatives. (**A**) The chemical structure of steviol, isosteviol, dihydroisosteviol, 16-oxime isosteviol, and CFTR_inh_-172. MDCK cells were seeded in 96-well plates, and incubated with the indicated concentrations of steviol, isosteviol, dihydroisosteviol, and 16 oxime isosteviol. Cell viability was assessed by MTT assay after 24 h (**B**) and 72 h (**C**) of incubation. Results were expressed as mean of % control±SE (n=3, P0.01).

Before proceeding with the assay of cyst formation and growth, MDCK cell viability was evaluated in the presence of 50, 100, and 200 µM steviol and its derivatives, isosteviol, dihydroisosteviol, 16-oxime isosteviol (Fig. 1A) using MTT assay. After 24 h, none of the compounds affected MDCK cell viability, but at 72 h isosteviol and 16-oxime isosteviol at the highest dose (200 µM) reduced cell viability by 23.9±1.8% and 24.2±0.8%, respectively (Fig. 1B and Fig. 1C). Thus, all subsequent experiments were conducted using steviol and derivatives at the concentration of 100 µM.

**Figure 2:**
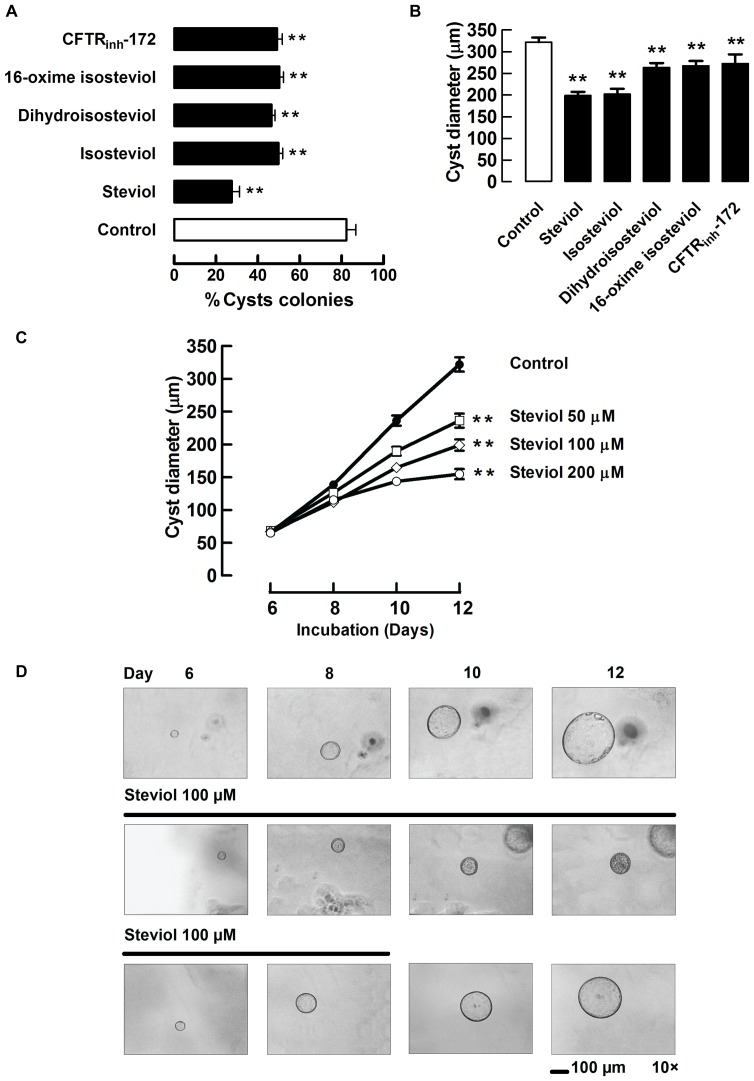
Effect of steviol and its derivatives on cyst progression in MDCK cyst model. (**A**) Inhibitory effect of steviol and its derivatives on MDCK cyst formation. MDCK cysts were incubated with 100 µM of steviol and its derivatives in media containing forskolin (10 µM) after cell seeding on day 0 onward. The graph represents percent of cyst colonies at day 6 after MDCK cell seeding in the absence (control) and presence of all compounds (mean±SE; *n* = 4 wells/condition; **P<0.01 compared with control). (**B**) Inhibitory effect of steviol and its derivatives on MDCK cyst growth. The graph shows the outer cyst diameter at day 12 (mean±SE; *n* = 32−77 cysts; **P<0.01 compared with control). (**C**) Dose-response of effect of steviol on MDCK cyst growth. After cell seeding in 3D collagen gel for 6 days, media containing forskolin and steviol at doses of 50, 100, and 200 µM were added to the MDCK cells from day 6 onward. Results were shown as mean value of cyst diameter at days 6, 8, 10, and 12 (*n* = 43−77 cysts; **P<0.01 compared with control). (**D**) Representative light micrographs show MDCK cyst growth in 3D collagen gel after seeding of MDCK cells for 6 days. Three independent experiments were performed. Forskolin (10 µM) without (**2D, top**) or with steviol (100 µM) (**2D, middle, bottom**) was added to the culture medium at day 6. To test for reversibility, steviol was removed at day 9 (**2D, bottom**). Scale bar = 100 µm; magnification = ×10.

To determine the effects of steviol and derivatives (isosteviol, dihydroisosteviol and 16-oxime isosteviol) on cyst formation in an *in vitro* model of PKD, MDCK cells seeded in collagen gel were exposed to 100 µM steviol, its 3 derivatives and 10 µM CFTR_inh_-172 (a CFTR inhibitor) [28] in the presence of 10 µM forskolin-containing media. At day 6, the number of cysts (outer diameter >50 µm) and non-cyst colonies were counted. The percent of cyst colonies in the control cell group was 82.2±4.5%, whereas the percents of cyst colonies in cells treated with steviol, isosteviol, dihydroisosteviol, 16-oxime isosteviol and CFTR_inh_-172 were 27.5±3.6%, 49.8±1.9%, 46.5±1.6%, 50.2±1.9%, and 49.2±2.4%, respectively (Fig. 2A). For determination of the inhibitory effect on cyst growth, steviol, the 3 derivatives (all at 100 µM) and CFTR_inh_-172 (at 10 µM) were added at day 6 to the forskolin-treated MDCK cells. At day 12, cyst diameters were measured and compared to those of the control group. The average cyst size was found to be decreased by 38.2%, 37.2%, 18.0%, 16.8%, and 15.3% with steviol, isosteviol, dihydroisosteviol, 16-oxime isosteviol and CFTR_inh_-172 treatments, respectively (Fig. 2B). In short, the average cyst diameters at day 12 of all treatment groups were significantly smaller than that of the control group. Taken together, these results indicated that steviol and its derivatives inhibited both cyst formation and growth in the MDCK cyst model and, among the compounds tested, steviol had the greatest effect. Therefore, only steviol was selected for further studies.

The inhibitory effect of steviol on cyst growth showed dose-dependency over the concentration range of 50 to 200 µM (Fig. 2C). The reversibility of the effect of steviol (100 µm) was examined by removing it from the cyst culture at day 9 (Fig. 2D). The inhibitory effect of steviol on cyst growth appeared to be abolished following its removal (Fig. 2D).

### Effect of steviol on cell proliferation and apoptosis

**Figure 3:**
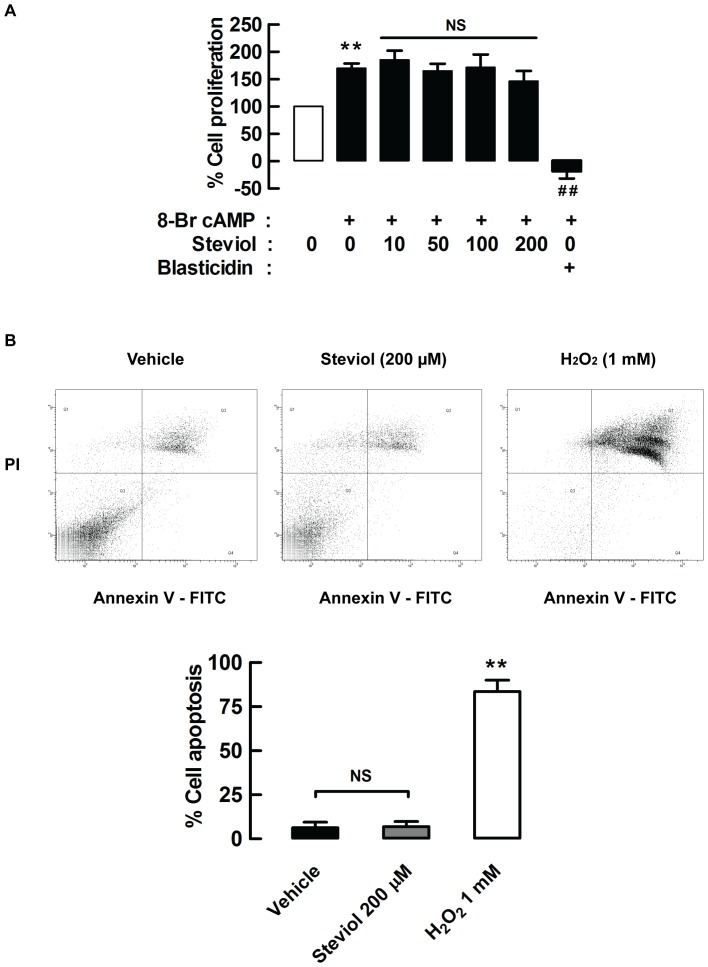
Effect of steviol on cell proliferation and apoptosis. (**A**) MDCK cell proliferation was assessed by BrdU cell proliferation assay. MDCK cells were seeded onto 96-well plates and grown for 24 h. The media containing 8-Br cAMP (100 µM) with or without steviol at doses of 10, 50, 100, and 200 µM was added and incubated for 24 h. BrdU was added at 18 h after addition of all compounds. The data represent percent of cell proliferation of MDCK cells treated with steviol at various concentrations. 20 µg/ml of blasticidin was used as positive control. Four independent experiments were done (mean of percent control±SE; *n* = 4, **P<0.01 compared with group of no cAMP treatment; *## P*<0.01 compared with cAMP treated group). (**B**) MDCK cell apoptosis was analyzed by flow cytometry. (**3B, top**) MDCK cell were incubated with steviol at a concentration of 200 µM for 24 h. Cells were stained with annexin V or propidium iodide. Apoptotic cells were localized in the lower right (early apoptosis) and upper right (late apoptosis) quadrants of the dot-pot graph using propidium iodide vs annexin V. 1 mM of hydrogen peroxide (H_2_O_2_) was used as positive control. (**3B, bottom**) The bar graphs represented percent of MDCK cell apoptosis of four independent experiments (mean percent control±SE; *n* = 4; **P<0.01 compared with control).

The inhibitory effect of steviol on MDCK cyst growth could involve suppression of cell proliferation or induction of apoptosis. Using a BrdU cell proliferation assay, we found that treatment with 8-Br cAMP (100 µM) for 24 hours stimulated MDCK cell proliferation and this was not affected by the presence of steviol (10, 50, 100, and 200 µM) (Fig. 3A). Similarly, steviol over the same concentration range did not induce apoptosis of MDCK cells as assessed using a flow cytometry assay (Fig. 3B). Therefore, inhibitory effect of steviol on cyst formation may result from inhibition of chloride secretion.

### Effect of steviol on chloride transport in MDCK cell monolayer

**Figure 4:**
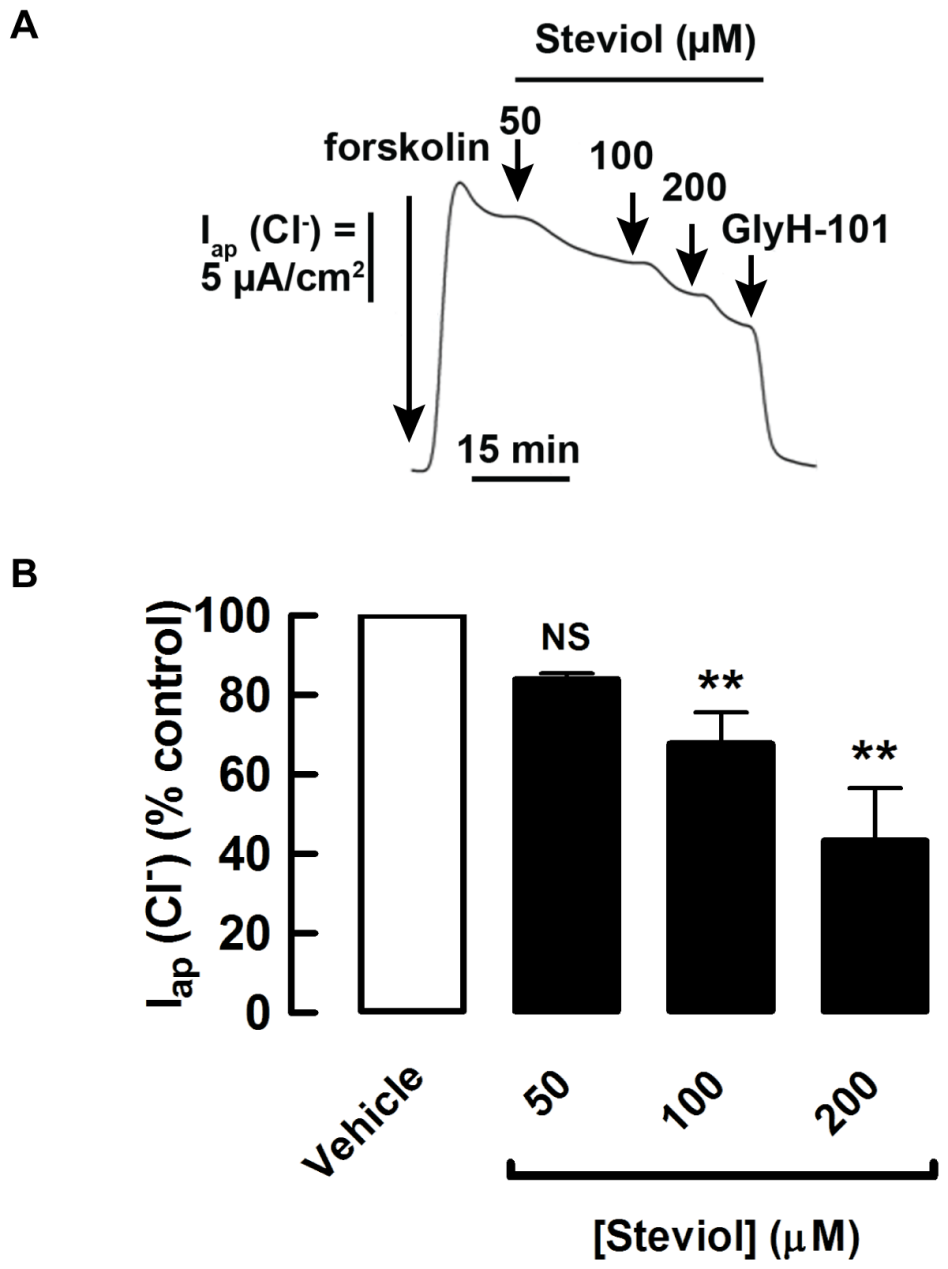
Inhibition by steviol of forskolin-stimulated apical chloride current. Under permeabilization conditions, MDCK cell monolayers were mounted in hemichambers filled with a chloride gradient buffer. (**A**) Representative currents of acute effect. Steviol at all doses was added to both apical and basolateral hemichambers after stimulation of the chloride current by forskolin (post-treatment) and the currents were recorded at 50, 100, and 200 µM of steviol. At the end of experiment, 50 µM of GlyH-101 was added. (**B**) Summary of the data for the acute effect of steviol on apical chloride current in basolaterally permeabilized MDCK cell monolayers. (4 separate experiments, mean±SE; *n* = 4; **P<0.01 compared with controls)

The acute effect of steviol on cAMP-activated chloride secretion of MDCK cells was determined by measuring apical chloride current in basolaterally permeabilized MDCK cells. Steviol (at concentrations of 50, 100, and 200 µM), when added to both apical and basolateral hemichambers after 15 min of forskolin (20 µM) stimulation, reduced the forskolin-stimulated apical chloride current in a dose-dependent manner, reaching 46.2±11.2% of the control current at 200 µM (Fig. 4).

**Figure 5:**
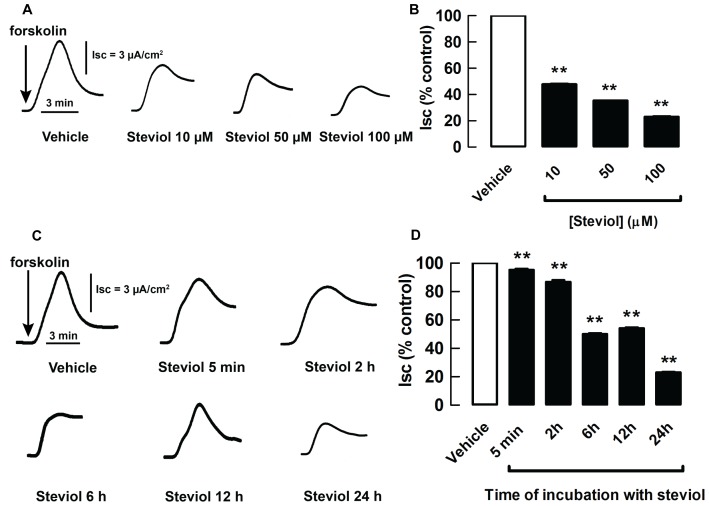
Inhibition by steviol of short-circuit current measurement in dose- and time-dependent manners. MDCK cell monolayer treated with steviol at the indicated concentrations or vehicle for 24 h were mounted in hemichambers filled with Krebs' buffer solution. After stimulation of current by forskolin, steviol inhibition was seen to have inhibited I_sc_ in a dose-dependent fashion. Representative tracings (**A**) and the summary of the data of 3–10 separate experiments (**B**) are shown (mean±SE; *n* = 3−10; **P<0.01 compared with controls). (**C**) Representative time-response tracings of short-circuit current measurements. Monolayers were pretreated with 100 µM of steviol for 5 minutes and 2, 6, 12, and 24 h. (**D**) Summary of the data from time-response experiments. (mean±SE; *n* = 7−16 separate experiments; **P<0.01 compared with controls)

**Figure 6:**
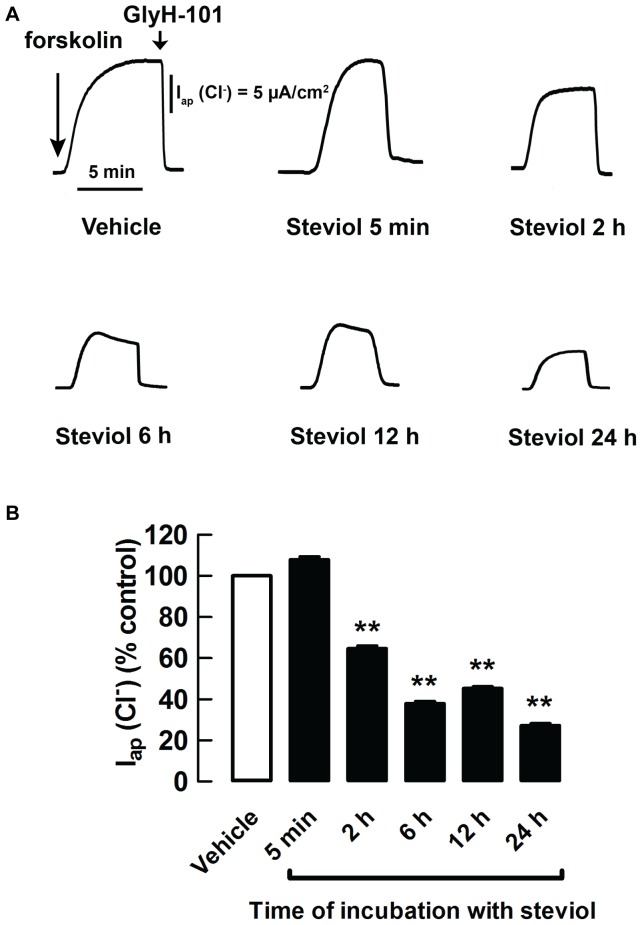
Steviol inhibits forskolin-stimulated apical chloride current in a time-dependent manner. After permeabilization of the basolateral membrane with amphotericin B, MDCK cell monolayers were mounted in hemichambers filled with a chloride gradient buffer. 20 µM of forskolin was added to stimulate apical chloride current. (**A**) Representative tracings of chronic effect (pretreatment with steviol for 5 minutes, 2, 6, 12, and 24 h) were shown. At the end of the experiment, 50 µM of Gly-H101 was added. (**B**) Summary of the data for the chronic effect of steviol on apical chloride current in permeabilized MDCK cell monolayers (mean±SE; *n* = 5−11 separate experiments; **P<0.01 compared with controls)

The time response and the prolonged effect of steviol on cAMP-activated chloride secretion were investigated using short-circuit current measurements in MDCK cell monolayers. Incubation of the cells for 24 h with 10–100 µM steviol resulted in reduction in short-circuit current (I_sc_) in a dose-dependent manner (Fig. 5A and 5B). When MDCK cell monolayers were treated with 100 µM steviol at various durations from 5 min up to 24 h, the significant reduction in I_sc_ was observed after 2 h of incubation. The effect became more pronounced at longer periods of incubation (Fig. 5C and 5D). Similar results were obtained for the apical chloride current measured in MDCK cell monolayers with amphotericin-B-permeabilized basolateral membranes in the presence of a basolateral-to-apical chloride gradient (Fig. 6). These sets of experiments suggested that steviol reduced CFTR expression in MDCK cells.

### Effect of steviol on CFTR protein expression in MDCK cells

**Figure 7:**
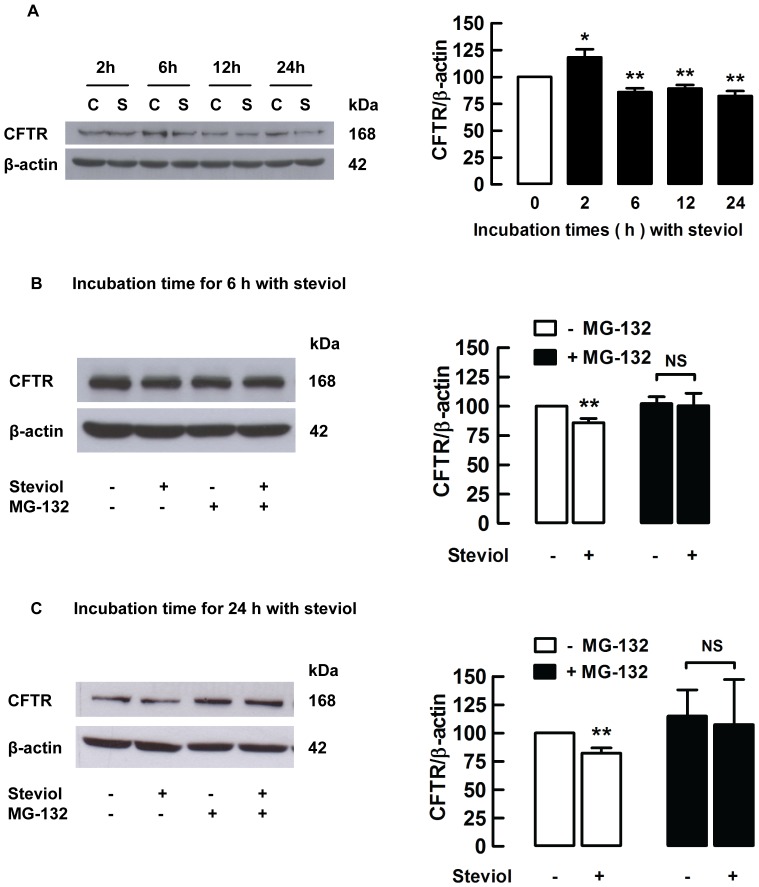
Steviol inhibits CFTR protein expression through proteasome pathway in MDCK cells. (**A**) MDCK cells were seeded in 6-well plates and grown for 48 h. They were incubated with either DMSO (C) or 100 µM of steviol (S) for 2, 6, 12, or 24 h and were blotted with antibodies to CFTR or β-actin. (**7A, left**) Band intensity of indicated protein expression. (**7A, right**) Densitometric analysis of the bands. CFTR expression is normalized to β-actin shown as bar graphs (mean percent of control±SE; *n* = 5−7 independent experiments; **P<0.01 compared with control). For proteasome pathway determination, western blots of MDCK cells were incubated with or without 50 µM of MG-132 for 1 h before treatment with DMSO (control) or 100 µM of steviol (experimental) for 6 h (**7B, left**) and 24 h (**7C, left**). For densitometric analysis, the values are represented as the ratio of CFTR/β-actin for 6 h (**7B, right**) and 24 h (**7C, right**) normalized to 100% of control group (mean percent of control±SE; *n* = 6−7 independent experiments; **P<0.01 compared with control).

To confirm that steviol reduced CFTR expression level in MDCK cells, MDCK cells were cultured in the presence of 100 µM steviol for 2–24 h and the levels of CFTR were determined using western blotting. Reduction in total CFTR content in MDCK cells was significantly reduced by 14.1±3.6% compared to control at 6 h of exposure to steviol and by 17.7±4.5% after 24 h (Fig. 7A). Previous studies have shown that a decrease in CFTR level could be via proteasome degradation pathway [29], [30]. In order to demonstrate that steviol employs this mechanism to reduce CFTR expression in MDCK cells, confluent MDCK cells were incubated with 50 µM proteasome inhibitor, MG-132, for 1 h before treatment with 100 µM steviol for 6 and 24 h, respectively. The results showed that an inhibitory effect of steviol on CFTR levels was abolished by treatment with MG-132 (Fig. 7B and Fig. 7C).

**Figure 8:**
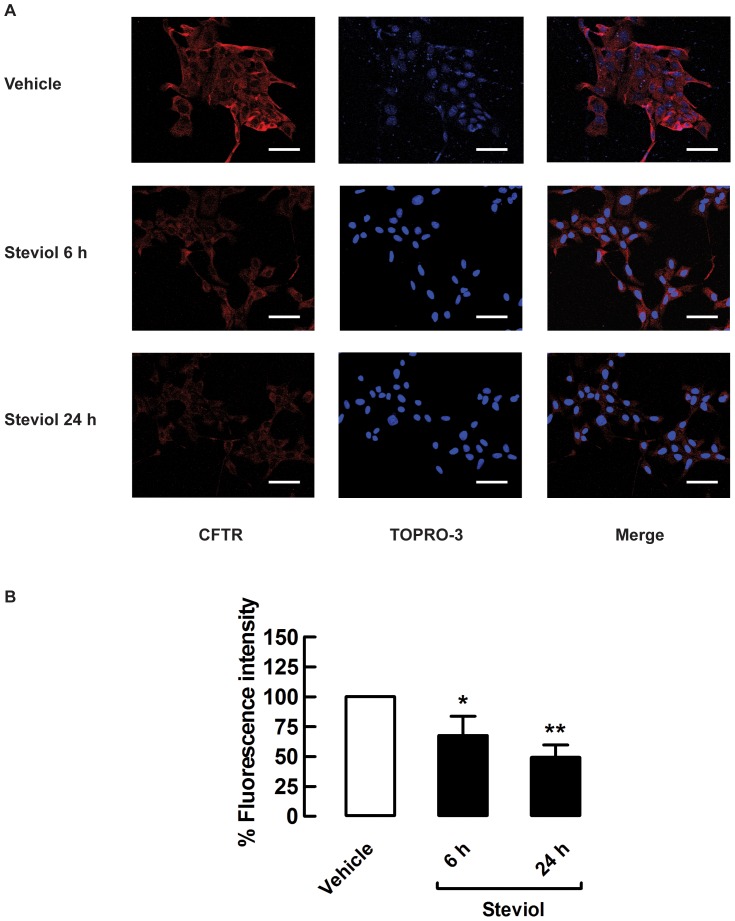
Steviol inhibition of CFTR membrane protein expression in MDCK cells. (**A**) Representative immunofluorescence images of CFTR (red), TOPRO-3-lebeled nuclei (blue) and merged images (*n* = 3). Scale bar = 50 µm; magnification = ×40. (**B**) Mean fluorescence intensity in MDCK cell after treatment with DMSO (vehicle) or 100 µM steviol (experimental) for 6 h and 24 h. The values are shown as percent fluorescence intensity (35 random regions of interest; mean percent of control±SE, *P<0.05, **P<0.01 compared with control).

In addition, determination of CFTR localization by immunofluorescence in intact cells indicated that 100 µM steviol decreased CFTR membrane expression by 32.4±16.0% and 50.8±10.4% compared to control at 6 h and 24 h of incubation, respectively (Fig. 8A and 8B). These findings are in agreement with those of the western blot analysis.

## Discussion

Cyst enlargement in PKD involves two pathogenic mechanisms, namely, increased epithelial cell proliferation and enhanced transepithelial fluid secretion [2], [4]. An increase in intracellular cAMP has been shown to stimulate cell proliferation through the MEK/ERK pathway and to activate CFTR-mediated chloride and fluid secretion into the cyst lumen [6], [7],[31]. Accumulation and expansion of cysts disrupt the normal function of renal parenchyma. Inhibition of CFTR by small molecule CFTR inhibitors, such as thiazolidinone and hydrazide-containing compounds, has been shown to slow cyst progression in both *in vitro* and *in vivo* models of PKD [21], [32].

The present study clearly demonstrated that the plant-derived sweetener steviol and its derivatives, isosteviol, dihydroisosteviol, 16-oxime isosteviol, at non-toxic levels (100 µM) were able to retard both cyst formation and enlargement in an *in vitro* MDCK cell model of PKD. From the chemical structures of steviol and three types of isosteviol studied, we can conclude that core structure of steviol rather than isosteviol with the presence of OH group on C-13 position may be associated with good inhibitory effect on renal cyst growth. The underlying mechanism was, in part, by direct inhibition of CFTR chloride channel activity and through reduction of CFTR expression via proteasome-mediated degradation. This notion was supported by the observation that the thiazolidinone CFTR_inh_-172, an allosteric blocker of the CFTR chloride channel, exerted inhibitory effects on MDCK cyst formation and growth (Fig. 2 and [21]). Moreover, previous studies revealed that the commonest cystic fibrosis mutation F508del, which prevents the plasma membrane expression of CFTR, slowed MDCK cyst enlargement by inhibiting CFTR-mediated fluid accumulation within the cyst lumen [33]. In addition, pioglitazone, a peroxisome proliferator-activated receptor γ (PPARγ) agonist inhibited vasopressin-induced chloride secretion through reducing CFTR mRNA levels in MDCK-C7 cell line [34] and suppressed cyst progression by decreasing apical CFTR expression in PCK rodent model of PKD [35].

Our previous study showed that 100 µM steviol inhibits apical chloride current in human colonic epithelial cells (T84 cells) by ∼50% [18], whereas in the present study it could only reduced forskolin-induced apical chloride current by 25% in MDCK cell monolayer. Based on the fact that dihydroisosteivol, a steviol derivative, inhibited CPT-cAMP-activated apical chloride current in basolaterally permeabilized T84 cells without the effects on intracellular cAMP levels [18] and steviol also inhibited forskolin-stimulated apical chloride current in the present study (Fig. 4), we speculate that steviol might inhibit CFTR directly as well. Therefore, one explanation for discrepancy between CFTR inhibition potencies in T84 and MDCK cells is the differences in steviol's binding sites between human CFTR expressed in T84 cells and canine CFTR expressed in MDCK cells. In support of this notion, the amino acid sequences in the membrane-spanning domains of CFTR in human, mouse, rat, and dog, are different [36], [37]. CFTR inhibitors, such as CFTR_inh_-172, glibenclamide and GlyH-101 have been found to exert their inhibitory effects differently on several CFTR orthologs because of differences in inhibitor-binding sites for each compound [38].

CFTR expressed at the plasma membrane has a short half-life, approximately 12 to 24 h [39], and hence an increase in the rate of CFTR degradation would have a significant effect on apical chloride secretion. Degradation of CFTR can occur via two different processes: an ER-associated degradation (ERAD) process involving a ubiquitin-proteasome pathway that degrades immature CFTR protein during biosynthesis, and a lysosomal pathway that destroys mature CFTR [40]. It has been shown that a cysteine string protein, a J-domain-containing protein involved in stimulated exocytosis, plays an important role in promoting proteasomal degradation of immature CFTR by increasing the interaction of immature CFTR with the c terminus of heat shock protein-70-interacting protein and thereby enhancing CFTR ubiquitylation [41].

We found that steviol effect on total CFTR protein expression was observed at 6 h, and such effect was sustained at the same level at later time points (12 h and 24 h). This finding suggests that steviol induced the reduction in CFTR protein expression via mechanisms involving modulation of CFTR trafficking and/or CFTR degradation. Besides, we found that pretreatment of the MDCK cells with MG-132 prior to exposure to steviol could completely prevent steviol-reduced CFTR expression, indicating that steviol reduces CFTR expression by promoting proteasome-mediated degradation of immature CFTR. However, it should be mentioned that MG-132 not only inhibits proteasomal activity, but also produces reactive oxygen species (ROS). Previous studies showed that prolonged treatment (24 h) of MG-132 at dose of 1–30 µM induces apoptosis via formation of ROS in several cancer cell types [42], [43]. It was also reported that incubation with 25 µM of MG-132 for 4 h prevented misfolded CFTR degradation in CHO cells expressing GFP tagged F508del-CFTR [44]. In addition, inhibition of proteasome-mediated CFTR degradation by MG-132 (10 µM, 1 h incubation) could prevent internalization and increased apical stability of mutant CFTR in human airway epithelial cells [45]. These studies indicate that prolonged incubation of MG-132 caused ROS production, whereas a short period of MG-132 treatment could inhibit CFTR degradation in bronchial cell lines. Therefore, incubation with 50 µM MG-132 for 1 h in our study was likely to inhibit proteasomal activity in MDCK cells without enhancing ROS. However, it should be cautioned that oxidative stress might interfere with the result obtained in the studies using MG-132. In addition, we found that steviol reduced the amount of CFTR protein expressed in the plasma membrane of MDCK cells by ∼32%, an extent which was much higher than its effect on total CFTR protein observed by western blot analysis (14% at 6 h after incubation with 100 µM steviol. Therefore, our results indicate that, in addition to its direct effect on CFTR channel activity, steviol reduces CFTR-mediated chloride transport in MDCK monolayers by promoting degradation of immature CFTR proteins and modulating plasma membrane turnover of CFTR. Steviol may also target CFTR maturation. However, further studies are needed to provide insight into the detailed mechanisms by which steviol reduces CFTR expression.

The inhibitory effects of steviol and its three derivatives on cyst progression in the MDCK cyst model correlate well with previous studies using other compounds (viz. thiazolidinone and hydrazide-containing CFTR inhibitors) in a mouse model of PKD [32]. Therefore, our results from MDCK cyst model suggested the possibility of using steviol to inhibit cyst expansion in an *in vivo* PKD models. Nonetheless, future studies using rodent models of PKD are required to evaluate the therapeutic potential of steviol and its derivatives in the treatment of PKD. Interestingly, it is estimated that administration of 5 mg/kg BW per day of stevioside results in a plasma concentration level of steviol of approximately 20 µM if stevioside is completely converted to steviol [46]. This plasma concentration is lower than the minimum concentration (50 µM) required to inhibit MDCK cyst growth. Pharmacokinetic studies of stevioside have indicated that a major metabolite of stevioside consumption (steviol glucuronide) is removed from the body by urinary excretion [47]. Thus, it is possible that steviol could reach cystic lesions in the kidney. However, stevioside consumption would have to be higher than 5 mg/kg BW per day to obtain enough steviol for therapeutic purposes. The Joint FAO/WHO Expert Committee on Food Additives has recommended that stevioside is safe and has no adverse effect when taken at doses of 4 mg/kg BW per day [48].

## Conclusions

In summary, we found that pharmacological concentrations of steviol retarded cyst progression in an *in vitro* MDCK cell model, in part, by reducing CFTR expression levels via proteasome-mediated CFTR degradation. These results indicate that steviol and related compounds represent promising natural plant-based drug candidates for treatment of polycystic kidney disease.
